# Workplace Interventions Targeting Mental Health Literacy, Stigma, Help-Seeking, and Help-Offering in Male-Dominated Industries: A Systematic Review

**DOI:** 10.1177/15579883241236223

**Published:** 2024-04-06

**Authors:** Emilie Roche, Noel Richardson, Jack Sweeney, Shane O’Donnell

**Affiliations:** 1 The National Centre for Men’s Health, Department of Health and Sports Sciences, South East Technological University, Carlow, Ireland

**Keywords:** men, mental health, workplace, male-dominated industries, psychosocial intervention

## Abstract

Mental ill-health and suicide represent a significant proportion of the burden of global disease among men. Connell’s relational theory of masculinities provides a useful framework to explore how mental health literacy, mental health stigma, and delayed help-seeking and help-offering behaviors are associated with mental ill-health among men, particularly within male-dominated industries. To address the high incidences of mental ill-health in male-dominated industries, several workplace interventions targeting these outcomes have been implemented. No review to date has examined the current state of evidence for these interventions or identified the behavior change techniques used. This review was restricted to empirical, quantitative research reporting on psychosocial interventions targeting mental health literacy, stigma, and help-seeking and help-offering behaviors in male-dominated industries. Quality appraisal was completed using the Effective Public Health Practice Project and a narrative synthesis was conducted. Twelve articles were included for review which reported on four distinct interventions. The methodological quality of two articles was strong, three moderate and seven weak. The strongest evidence of intervention effects related to mental health literacy and help-seeking intentions. There was less evidence relating to help-offering and help-seeking behaviors and mental health stigma. Sixteen behavior change techniques were identified across interventions that are discussed in relation to the wider men’s health literature. The evidence on psychosocial interventions in male-dominated industries is limited due to methodological and conceptual issues. Recommendations for future research include standardized reporting of intervention descriptions, the use of theory to guide intervention development, and utilizing validated and reliable outcome measures.

## Introduction

Men’s mental health has been labeled a “silent epidemic” (Bilsker & White, 2011; Whitley, 2018). Common mental health problems such as anxiety and depression are cited as significant contributors to the global burden of disease among men (Baker et al., 2014; Rehm & Shield, 2019). Arguably, the real extent of the issue is best highlighted by the disproportionate suicide rates among men in high-income countries (World Health Organization [WHO], 2021). Despite this, rates of suicidal ideation, self-harm, suicide attempt, anxiety, and depression are lower in males compared with females (Boyd et al., 2015; Girgus et al., 2017; Miranda-Mendizabal et al., 2019). However, this does not necessarily indicate better mental health outcomes among men but rather reflects their lower likelihood to engage with mental health supports or to be formally diagnosed with a mental health problem. A body of evidence has drawn on Connell’s relational theory of masculinities to explore the interconnectivities between men and mental ill-health (Apesoa-Varano et al., 2018; Connell & Messerschmidt, 2005; Mac an Ghaill & Haywood, 2012; O’Donnell & Richardson, 2020; Oliffe et al., 2016; Richardson et al., 2023; Scourfield, 2005). Adherence to masculine ideologies such as stoicism, self-reliance, competitiveness, and desire for control—coupled with the suppression of emotions and vulnerability—is reported to influence men’s health practices and illness experiences (Coleman, 2015; Möller-Leimkühler, 2003; Payne et al., 2008; Pirkis et al., 2017).

It is against this backdrop that poor health literacy, increased mental health stigma, and delayed help-seeking are implicated as key challenges to men’s mental health (Cotton et al., 2006; McKenzie et al., 2022; Möller-Leimkühler, 2002). Lack of engagement with health-care services may result from lower levels of competence around when to seek help with previous research highlighting an association between conformity to masculine norms and lower health literacy (Milner et al., 2019). This may affect men’s ability to recognize mental health disorders which may contribute to increased stigma and delayed help-seeking (Cotton et al., 2006; McKenzie et al., 2022).

Work is recognized as a key setting in which masculine stereotypes are enacted and reproduced (Connell, 2012). This may be particularly pronounced in male-dominated industries that are reported to have a “macho” workplace culture (Milner, Law, et al., 2018). Men in male-dominated industries (>70% male employees) are reported to have a higher risk of suicide compared with the general working population (Milner, Kavanagh, et al., 2018; Tyler et al., 2024). Employees in male-dominated industries are less likely to seek help from a professional during times of emotional distress (Milner, Kavanagh, et al., 2018), have lower health literacy (Milner et al., 2020), and experience significant mental health-related stigma (Seaton et al., 2019). This resulted in the development of interventions to increase mental health literacy, reduce stigma, and improve help-seeking and help-offering behaviors in these industries (Kennedy et al., 2020; King et al., 2018; Milner et al., 2020; Seaton et al., 2019). Previous systematic reviews conducted in male-dominated industries have focused on health and well-being more broadly (Hulls et al., 2022), on organizational level interventions (Greiner et al., 2022), on specific occupations within male-dominated industries (Younker & Radunovich, 2022), or on depression and anxiety outcomes (Roche et al., 2016). No systematic review to date has shed light on the current state of evidence of workplace psychosocial interventions on increasing mental health literacy, reducing stigma, or improving help-seeking or help-offering behaviors in male-dominated industries.

A key step in the development of an effective behavior change intervention is to report the observable and replicable components that elicit change, commonly referred to as behavior change techniques or BCTs (Michie et al., 2013). While previous research has highlighted how to design interventions to engage men around their health using gender-responsive approaches (Galdas et al., 2023; Struik et al., 2019), little research to date has identified the mechanism of change, or BCTs, in such interventions. Previous reviews have synthesized BCTs within interventions targeting help-seeking among men (Sagar-Ouriaghli et al., 2019) and within workplace wellness programs more generally (De Korte et al., 2018; Ryan et al., 2021). However, no research to date has synthesized BCTs within interventions in male-dominated industries. This is crucial if we are to identify, replicate, and implement these intervention components to better understand their effects (Michie et al., 2013) within the context of a male-dominated work environment. Therefore, the aim of this review is to shed light on the current state of evidence of psychosocial interventions targeting mental health literacy, stigma, and help-seeking and help-offering behaviors in male-dominated industries and to identify the BCTs used in these interventions.

## Method

This systematic review is reported using the PRISMA (Preferred Reporting Items for Systematic Reviews and Meta-Analyses) guidelines and was registered on PROSPERO (No. CRD42022340520). A systematic search was conducted on July 12, 2022, and again on August 21, 2023, using the electronic databases MEDLINE, PsycINFO, Scopus, Web of Science, and CINAHL. Search terms were a mixture of MeSH and keywords informed by previous reviews (Hulls et al., 2022; Roche et al., 2016) and were refined using an iterative process (see Supplementary File 1).

The search was restricted to empirical, quantitative studies of workplace interventions in male-dominated industries that reported on outcomes relating to mental health literacy, mental health stigma, and/or help-seeking or help-offering. A workplace intervention was defined as the delivery of an activity or activities in the workplace, designed to improve health status (O’Cathain et al., 2019). The male-dominated industries that were included in this review were the construction, manufacturing, mining, utilities, transport, agriculture, and information technology sectors. This was informed by previous reviews that have classified a male-dominated industry as consisting of at least a 70% male workforce (Hulls et al., 2022; Lee et al., 2014; Roche et al., 2016). Emergency workers (police, fire, emergency medical response, search and rescue) and members of the defense forces were excluded due to their disproportionate exposure to traumatic events that may require more targeted interventions (WHO, 2022). This is in line with previous reviews that have focused on these groups specifically (Claringbold et al., 2022) or that have excluded them within the context of male-dominated industries (Hulls et al., 2022; Roche et al., 2016).

Mental health literacy was defined as knowledge and beliefs about mental disorders that aid in their recognition, management, and prevention (Jorm, 2012). Mental health stigma was stratified into: (a) self-stigma—internalized negative attitudes toward one’s own mental health illness; (b) personal stigma—negative attitudes toward groups with mental illness; and (c) public stigma—perceived negative attitudes held by the public toward people with a mental illness (Schnyder et al., 2017). Help-seeking and help-offering included any outcomes relating to attitudes, willingness, intentions, or behaviors associated with seeking help for mental ill-health or supporting others experiencing mental ill-health.

Titles and abstracts of identified studies were screened independently against the eligibility criteria by two reviewers (E.R. and J.S.). Full-text screening was then conducted independently by two reviewers (E.R. and N.R.). Discrepancies were arbitrated by a third reviewer (S.O.D). Finally, hand-searching was conducted to identify eligible papers that may have been missed through the online database search. Data were extracted using a data extraction tool that included: (a) general study and participants’ characteristics (study design, aim; country, duration; number of participants; age; sex; population; industry); (b) intervention characteristics (intervention function, content, mode of delivery, duration, BCTs; culturally adapted elements); and (c) study outcomes. Intervention functions constituted nine broad categories of means by which an intervention can change behavior (Michie et al., 2014). Data extraction was performed independently by two reviewers (E.R. and S.O.D). Quality appraisal was conducted independently by two reviewers (E.R. and S.O.D) using the Effective Public Health Practice Project Quality Assessment Tool for Quantitative Studies (Thomas et al., 2004). This tool has been recommended for use when assessing public health interventions with varying study designs (Deeks et al., 2003). Study quality was assessed across seven domains with each domain being rated as strong, moderate, or weak. A global score was then derived for each study including strong (no weak ratings), moderate (one weak rating), and weak (two or more weak ratings). Due to the heterogeneity of interventions and outcomes, a narrative synthesis was conducted.

## Results

### Study and Participant Characteristics

**Figure 1.**
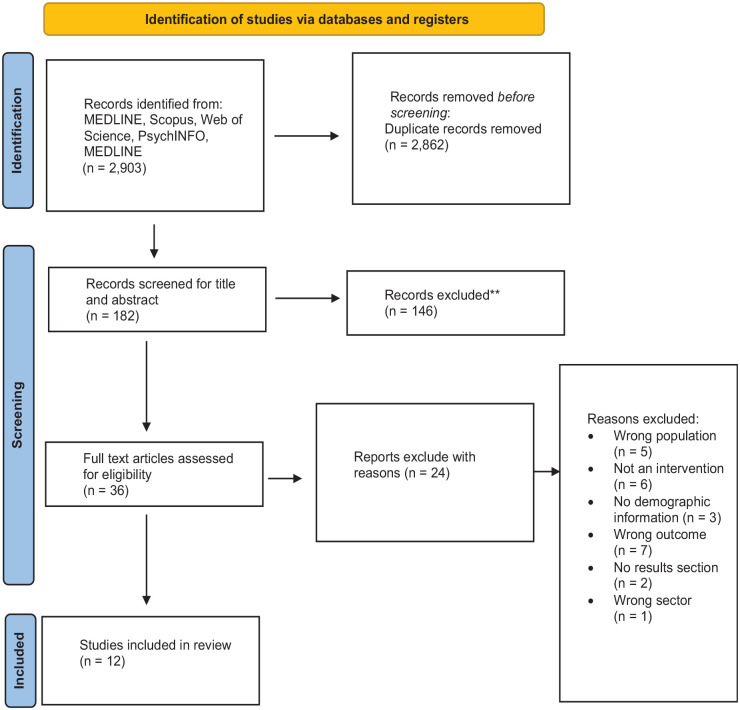
PRISMA Flow Diagram *Note*. PRISMA = Preferred Reporting Items for Systematic Reviews and Meta-Analyses.

Twelve quantitative articles were included in this review following the screening process (see Figure 1). This included four RCTs (Gast et al., 2022; King et al., 2023; Milner, Law, et al., 2018; Milner et al., 2019); one clinical controlled trial (Tynan et al., 2018); three one group pre/post designs (King et al., 2018; Ross, Caton, Gullestrup, & Kõlves, 2020; Sage et al., 2016); two interrupted time series designs (Ross, Caton, Mathieu, et al., 2020; Schwarz et al., 2019); one non-equivalent controlled pre/post design (Gullestrup et al., 2011); and one repeated cross-sectional survey (Sayers et al., 2019). The study duration period ranged from 6 weeks to 31 months. Most studies were conducted in Australia and in the construction sector. There was a pooled total of 40,748 participants across all studies. Nine articles reported the sex of participants of which 72% were male with four studies consisting of all male participants.

### Quality Appraisal

Two articles were rated as strong (Milner, Law, et al., 2018; Milner et al., 2019), three as moderate (Gast et al., 2022; King et al., 2018; Ross, Caton, Gullestrup, & Kõlves, 2020), and seven as weak (Gullestrup et al., 2011; King et al., 2023; Ross, Caton, Mathieu, et al., 2020; Sage et al., 2016; Sayers et al., 2019; Schwarz et al., 2019; Tynan et al., 2018). The most common methodological limitations identified within the articles related to selection bias, the reliability and validity of the data collection methods, and the adequacy of controlling for confounders. Table 1 provides a more detailed description of the quality appraisal process.

**Table 1.** The Effective Public Health Practice Project (EPHPP) Checklist Criteria for Each Study

Author and year of publication	Selection bias	Study design	Confounders	Blinding	Data collection methods	Withdrawals and dropout	Global score
Gullestrup et al., 2011	+	-	-	+	-	+	Weak
King et al., 2023	++	++	-	+	-	-	Weak
King et al., 2018	+	+	++	+	-	+	Moderate
Milner et al., 2018	++	++	++	++	++	+	Strong
Milner et al., 2019	++	++	++	++	++	+	Strong
Ross, Caton, Gullestrup, & Kõlves, 2020	+	+	++	+	-	+	Moderate
Ross, Caton, Mathieu, et al., 2020	+	+	-	+	-	-	Weak
Sage et al., 2016	-	+	-	+	-	-	Weak
Sayers et al., 2019	-	-	++	+	+	+	Weak
Tynan et al., 2019	+	+	-	+	-	-	Weak
Gast et al., 2022	-	++	++	+	++	++	Moderate
Schwarz et al., 2019	-	+	++	+	++	-	Weak

- = weak; + = moderate; ++ = strong.

### Intervention Characteristics

Six articles evaluated different iterations of a multilevel, peer-based suicide prevention program that was delivered in the construction (Gullestrup et al., 2011; King et al., 2018; Ross, Caton, Mathieu, et al., 2020), mining (Sayers et al., 2019; Tynan et al., 2018), and energy (Ross, Caton, Gullestrup, & Kõlves, 2020) sectors. Five articles evaluated the general awareness training (GAT) component of the intervention (Gullestrup et al., 2011; King et al., 2018; Ross, Caton, Gullestrup, & Kõlves, 2020; Ross, Caton, Mathieu, et al., 2020; Tynan et al., 2018); one article evaluated a shortened version of the GAT component called “MATES awareness training” (MAT) (Ross, Caton, Mathieu, et al., 2020); three articles evaluated the SafeTalk and ASIST components of the interventions (Gullestrup et al., 2011; Sayers et al., 2019; Tynan et al., 2018); one article evaluated a manager training component of the intervention (Tynan et al., 2018); one article evaluated the impact of three iterations: GAT; Gatekeeper “Connector” training; and SafeTalk and ASIST (Sayers et al., 2019); and one evaluated the impact of the GAT in conjunction with a mobile app (MATESmobile) (King et al., 2023). Two articles reported on a brief contact intervention conducted in the construction industry that sent hyperlinks with information on stigma and mental health literacy via text message (Milner, Law, et al., 2018; Milner et al., 2019). Two articles reported on stress management and stigma reduction manager training in the industrial sector (Gast et al., 2022; Schwarz et al., 2019). Finally, one article reported on a trauma-risk management (TRiM) program in the transport sector (Sage et al., 2016). Intervention duration ranged from 15 min to 2 days and the majority were delivered face-to-face. Only two of 12 articles reported on underpinning theories behind the interventions (Gast et al., 2022; Schwarz et al., 2019). See Supplementary File 2 for a more detailed description of study and intervention characteristics.

### Intervention Functions and BCTs

The most identified intervention functions were Education—increasing knowledge or understanding; Training—imparting skills; Modeling—providing an example for people to aspire to or imitate; and Persuasion—using communication to induce positive or negative feelings or stimulate action. The BCT “information about consequences” (health, emotional, or social and environmental) was identified in 10 articles primarily relating to raising awareness about the issues of mental ill-health, suicide, and/or post-traumatic stress disorder (PTSD); the impact of stigma; and the importance of social support, communication, and help-seeking (Gullestrup et al., 2011; King et al., 2018, 2023; Milner, Law, et al., 2018; Milner et al., 2019; Ross, Caton, Gullestrup, & Kõlves, 2020; Ross, Caton, Mathieu, et al., 2020; Sage et al., 2016; Sayers et al., 2019; Tynan et al., 2018).

“Information about antecedents”—defined as the emotions that precede or predict a behavior—was identified in all included articles primarily relating to improving knowledge about risk and protective factors and signs of stress, mental ill-health, PTSD, and/or suicidal behavior (Gast et al., 2022; Gullestrup et al., 2011; King et al., 2018, 2023; Milner, Law, et al., 2018; Milner et al., 2019; Ross, Caton, Gullestrup, & Kõlves, 2020; Ross, Caton, Mathieu, et al., 2020; Sage et al., 2016; Sayers et al., 2019; Schwarz et al., 2019; Tynan et al., 2018). The BCT “social support” related to providing information and/or facilitating discussion groups on where to seek-help; how to refer someone in distress; how to structure conversations around mental health and manage employees in distress; telephone support or counseling; encouraging people to seek or offer help; and providing psychological counseling to workers; was identified in 11 articles (Gast et al., 2022; Gullestrup et al., 2011; King et al., 2018, 2023; Milner, Law, et al., 2018; Milner et al., 2019; Ross, Caton, Gullestrup, & Kõlves, 2020; Ross, Caton, Mathieu, et al., 2020; Sayers et al., 2019; Schwarz et al., 2019; Tynan et al., 2018).

The BCTs “instruction on how to perform behavior,”“demonstration of behavior,” and “behavioral practice/rehearsal” often co-occurred and were identified in 10 articles (Gast et al., 2022; Gullestrup et al., 2011; King et al., 2018, 2023; Ross, Caton, Gullestrup, & Kõlves, 2020; Ross, Caton, Mathieu, et al., 2020; Sage et al., 2016; Sayers et al., 2019; Schwarz et al., 2019; Tynan et al., 2018). These BCTs related to role-play and demonstrating how to support employee mental health and how to refer someone in distress. The BCT “credible source” was identified in seven articles relating to program content being delivered by a facilitator with lived experience of mental ill-health (Gullestrup et al., 2011; King et al., 2018, 2023; Ross, Caton, Gullestrup, & Kõlves, 2020; Ross, Caton, Mathieu, et al., 2020; Sayers et al., 2019; Tynan et al., 2018). Finally, the BCT “prompts/cues” was present in two articles and related to regular SMS messages with links to learning materials around mental health” (Milner, Law, et al., 2018; Milner et al., 2019). See Supplementary File 2 for a more detailed description of intervention BCTs.

### Study Outcomes

Study outcomes were categorized and reported under the following headings: Mental health literacy; Stigma; Help-seeking; and Help-offering.

### Mental Health Literacy

Nine articles assessed different components of mental health literacy, which included myths and misconceptions concerning mental ill-health (Gast et al., 2022; Schwarz et al., 2019); recognizing the signs of distress (Ross, Caton, Gullestrup, & Kõlves, 2020; Ross, Caton, Mathieu, et al., 2020; Sage et al., 2016; Tynan et al., 2018); knowledge of available support services (Ross, Caton, Gullestrup, & Kõlves, 2020; Sage et al., 2016; Tynan et al., 2018); competence to engage with a distressed colleague and refer them to appropriate supports (Ross, Caton, Mathieu, et al., 2020; Sage et al., 2016; Tynan et al., 2018); and myths and misconceptions concerning suicide (Gullestrup et al., 2011; King et al., 2018, 2023; Ross, Caton, Gullestrup, & Kõlves, 2020; Ross, Caton, Mathieu, et al., 2020).

Two articles reported significant improvements in knowledge-related stigma using the mental health knowledge scale among managers in the industrial sector (Gast et al., 2022; Schwarz et al., 2019). These findings were observed in a pre/post-test design that were sustained at 3- and 12-month post-intervention (Schwarz et al., 2019) and were replicated in an RCT where significant improvements were observed among the intervention group compared with the control at 3 months post-intervention (Gast et al., 2022).

Four articles reported significant improvements in recognizing the signs of distress in a colleague (Ross, Caton, Gullestrup, & Kõlves, 2020; Ross, Caton, Mathieu, et al., 2020; Sage et al., 2016; Tynan et al., 2018). Two articles reported significant improvements in knowledge of available supports (Ross, Caton, Gullestrup, & Kõlves, 2020; Tynan et al., 2018), while one reported no significant difference (Sage et al., 2016). One article reported a significant increase in competence to discuss mental health with colleagues (Sage et al., 2016) while two reported increased knowledge of how to connect a colleague to supports (Ross, Caton, Mathieu, et al., 2020; Tynan et al., 2018).

Finally, five articles reported on mental health literacy concerning suicide prevention (Gullestrup et al., 2011; King et al., 2018, 2023; Ross, Caton, Gullestrup, & Kõlves, 2020; Ross, Caton, Mathieu, et al., 2020). These articles used ad hoc questions resulting in significant variance in the questions used to assess this concept. There were significant improvements relating to knowledge of suicide warning signs (Gullestrup et al., 2011; King et al., 2018; Ross, Caton, Gullestrup, & Kõlves, 2020; Ross, Caton, Mathieu, et al., 2020); the high incidence of suicide in male-dominated industries (Gullestrup et al., 2011; King et al., 2018; Ross, Caton, Gullestrup, & Kõlves, 2020); and that suicide is often an escape from pain rather than a wish to die (Gullestrup et al., 2011). One article reported a significant improvement in awareness that talking about suicide does not cause suicide (Ross, Caton, Mathieu, et al., 2020) while another article reported no significant improvement in this belief (King et al., 2018). Finally, King et al. (2023) reported no significant difference in suicide prevention literacy between a blended face-to-face and smartphone intervention and a face-to-face intervention only among construction workers in an RCT study.

### Stigma

Six articles reported on different components of stigma (Gast et al., 2022; Milner, Law, et al., 2018; Sage et al., 2016; Sayers et al., 2019; Schwarz et al., 2019; Tynan et al., 2018). One article assessed self-stigma (Milner, Law, et al., 2018); two assessed personal stigma (Gast et al., 2022; Schwarz et al., 2019); two assessed public stigma (Sayers et al., 2019; Tynan et al., 2018); and two assessed a combination of these constructs using ad hoc measures (Sage et al., 2016; Schwarz et al., 2019). There was no significant effect on the self-stigma of depression scale in an RCT (Milner, Law, et al., 2018) nor on ad hoc items regarding perceived treatment from line manager, personal shame, embarrassment, perceived weakness, or potential harm to career associated with mental health difficulties (Sage et al., 2016; Schwarz et al., 2019).

In relation to personal stigma, there were no statistical improvements reported on the social distance scale (Gast et al., 2022; Schwarz et al., 2019) or on the ad hoc items relating to shame and avoidance of those with mental health problems, and giving responsibility to colleagues with mental health problems (Sage et al., 2016; Schwarz et al., 2019). However, there was a significant positive improvement in ad hoc items relating to attitudes toward a colleague receiving mental health treatment (Sage et al., 2016).

There were mixed findings in relation to public stigma which appeared to be determined by the phrasing of the question and who might be perpetrating the stigma. There were significant changes to the belief that “friends,”“work colleagues,” and/or “supervisors” would treat a person with a mental health difficulty “fairly” (Schwarz et al., 2019) or that they would not treat such a person “differently” (Sayers et al., 2019). However, there were no significant changes to the belief that a person would not be “treated poorly” in the workplace more generally (Sayers et al., 2019; Tynan et al., 2018).

### Help-Seeking

Three articles reported on attitudes toward help-seeking (Ross, Caton, Gullestrup, & Kõlves, 2020; Ross, Caton, Mathieu, et al., 2020; Sage et al., 2016); four articles reported on intention to seek-help (King et al., 2023; Ross, Caton, Gullestrup, & Kõlves, 2020; Ross, Caton, Mathieu, et al., 2020; Sayers et al., 2019); and one article reported on incidences of help-seeking behavior (Tynan et al., 2018).

Three articles used ad hoc measures to assess attitudes toward help-seeking (Ross, Caton, Gullestrup, & Kõlves, 2020; Ross, Caton, Mathieu, et al., 2020; Sage et al., 2016). Two reported a significant change in general willingness to seek help (Ross et al., 2020; Ross, Caton, Gullestrup, & Kõlves, 2020), while the other reported a significant positive improvement in the belief that attending professional supports would remain confidential (Sage et al., 2016). However, the latter study observed no significant differences regarding trust in mental health professionals or the belief they would be allowed time away from work for treatment (Sage et al., 2016). This study had an adverse effect by increasing the belief that managers would discourage help-seeking from professional support.

Four articles reported on help-seeking intentions using the general help-seeking questionnaire (Ross, Caton, Gullestrup, & Kõlves, 2020; Ross, Caton, Mathieu, et al., 2020; Sayers et al., 2019; King et al., 2023). Three articles reported significant improvements regarding intention to seek-help from family, friends, a workmate, or a supervisor (Ross, Caton, Gullestrup, & Kõlves, 2020; Ross, Caton, Mathieu, et al., 2020; Sayers et al., 2019); intention to access support from helplines and “other sources” (Ross, Caton, Gullestrup, & Kõlves, 2020; Ross, Caton, Mathieu, et al., 2020), religious leaders, and partners (Ross, Caton, Mathieu, et al., 2020); an employee assistance program (Sayers et al., 2019); and a psychologist (Ross, Caton, Gullestrup, & Kõlves, 2020; Sayers et al., 2019). One article reported a significant difference in intention to seek help from a gatekeeper trained in suicide prevention among the intervention group exposed to a blended face to face and smart phone intervention compared with a face to face only control group; however, there was no significant difference for all other sources of support (King et al., 2023).

There were mixed findings relating to willingness to seek-help from more professional supports such as a doctor, mental health professionals, counselors, and/or “gatekeepers” on-site (Ross, Caton, Gullestrup, & Kõlves, 2020; Ross, Caton, Mathieu, et al., 2020; Sayers et al., 2019). Females had significantly greater intentions to seek help compared with males, higher levels of psychological distress was associated with lower intention to seek help, while older workers preferred more formal supports compared with younger workers (Ross, Caton, Gullestrup, & Kõlves, 2020; Sayers et al., 2019).

Finally, one article reported on incidences of help-seeking and observed no significant difference in incidences of help-seeking between three time points following delivery of a suicide prevention intervention (Tynan et al., 2018).

### Help-Offering

Four articles reported on willingness and competence to offer help (Ross, Caton, Gullestrup, & Kõlves, 2020; Ross, Caton, Mathieu, et al., 2020; Sage et al., 2016; Tynan et al., 2018). One study reported a significant improvement in willingness to offer help (Ross, Caton, Gullestrup, & Kõlves, 2020) while two others reported no significant improvement (Ross, Caton, Mathieu, et al., 2020; Sage et al., 2016). One article reported that employees were significantly more confident and competent to start a conversation around mental health post-intervention (Tynan et al., 2018).

## Discussion

This aim of this review was to shed light on the current state of evidence for workplace interventions targeting mental health literacy, stigma, and help-seeking and help-offering behaviors in male-dominated industries and to identify BCTs used within interventions. Overall, there was a distinct lack of experimental studies with longitudinal outcomes powered to show effect. Poor research design coupled with variance in sample size across included studies further undermined the findings, with only four RCTs included in the review. While it is promising to see the recent registration of RCTs (LaMontagne et al., 2022), more are needed to build the evidence base to further the field.

Like previous reviews, a notable finding was the absence of intervention content, logic models, and underpinning theory (Greiner et al., 2022; Hulls et al., 2022; Sagar-Ouriaghli et al., 2019), making it difficult to draw any firm conclusions regarding the effects of included interventions on mental health literacy, stigma, help-seeking, or help-offering. More than half of the included articles were rated as being of “weak” methodological quality and weak articles were more likely to report significant findings compared with articles that were rated as “moderate” or “strong” quality calling the validity of study results into question. The most common methodological issues are related to the reliability and validity of data collection methods, selection bias, and not controlling for confounders. Many studies were not explicit about the primary outcome under investigation, did not provide a clear construct definition, and used ad hoc questions to assess study outcomes despite the availability of well-established measures. The use of controlled robust studies with longitudinal outcomes that provide clear construct definitions that explicitly state the primary outcomes and utilize validated measures are recommended to further the evidence base in male-dominated industries.

Consistent reporting of interventions has been recommended to enhance intervention design (Conn & Groves, 2011) and the absence of underpinning theory in included interventions may be an explanatory factor for insignificant findings relating to complex behavioral outcomes like help-seeking. A focus on more theoretically driven interventions would ensure that all aspects of the help-seeking process (i.e., problem recognition and awareness of services) are targeted (Cornally & McCarthy, 2011). Existing intervention development frameworks such as the Medical Research Council guidance for complex interventions (Craig et al., 2008) or the Behavior Change Wheel (Michie et al., 2014) should be used to guide future interventions to ensure a robust rationale for interventions. It is recommended that future research utilizes a standardized approach to reporting intervention content such as the Template for intervention description and replication (TIDieR) Checklist (Hoffmann et al., 2014) that provides information on the rationale, theories, and goals of intervention elements. The Theory of Planned Behavior (Ajzen, 1991) may be a useful underpinning theory to guide interventions targeting help-seeking or help-offering.

Despite these limitations, there were some notable findings. Nine articles reported significant improvements in mental health literacy post-intervention, which related to myths and misconceptions concerning mental ill-health and suicide, knowledge of available support services, engaging with a distressed colleague, and referring them to appropriate supports (Gast et al., 2022; Gullestrup et al., 2011; King et al., 2018; Ross, Caton, Gullestrup, & Kõlves, 2020; Ross, Caton, Mathieu, et al., 2020; Sage et al., 2016; Sayers et al., 2019; Schwarz et al., 2019; Tynan et al., 2018). This is an important finding considering men’s reported lower levels of mental health literacy compared with females (Cotton et al., 2006; Oliffe et al., 2016) and the role of mental health literacy in early recognition of mental health disorders (Jorm et al., 1997). Most interventions targeting mental health literacy outcomes were through psychoeducational material—knowledge of signs, awareness of symptoms—which aligns with existing findings for effective strategies to improve mental health literacy within male-dominated industries (Lee et al., 2014). While these findings are promising, they should be interpreted in the context of improved mental health literacy being seen not as an end goal, but rather as a component part of a more complex process of promoting positive mental health overall (Jorm, 2019). Future research might consider longitudinal studies to investigate the extent to which an increase in mental health literacy results in an observable behavior change that benefits mental health outcomes.

There were no significant improvements for self-stigma or personal stigma using validated scales. The only improvements in stigma were observed among studies that used non-validated measures. Stigma is a complex phenomenon that encompasses deeply ingrained beliefs (Corrigan & Rao, 2013) that are likely to require more than a short-term intervention to bring about meaningful change. Stigma as a concept can be difficult to measure, and previously reported methodological issues in gauging the intervention effects of workplace stigma interventions (Hanisch et al., 2016) were also been borne out by this review. Despite insignificant findings, it remains important to address mental health stigma within male-dominated industries as the “macho” environment may perpetuate cultural masculine norms that affect how workers view mental health (Eyllon et al., 2020). More robust longitudinal studies are needed that utilize interventions of longer duration, clearly articulate the type of stigma being investigated, and use validated measurements to ensure construct validity.

There was evidence that a peer-based suicide prevention intervention increased help-seeking intentions, particularly from informal sources (Ross, Caton, Gullestrup, & Kõlves, 2020; Ross, Caton, Mathieu, et al., 2020; Sayers et al., 2019), aligning with some men’s preference to engage with mental health in more informal settings (O’Donnell & Richardson, 2018; Seaton et al., 2017). These findings contrast with a previous review highlighting a lack of evidence for psychoeducational interventions to bring about change to help-seeking intentions more broadly (Gulliver et al., 2012). However, this may be attributed to the co-production approach of the multimodal, peer-based suicide prevention intervention which complements existing findings around engaging men as equal partners in designing more acceptable health programs in male-dominated industries (Greiner et al., 2022).

While a reported increase in help-seeking intentions is promising, there were no significant intervention effects reported for actual help-seeking behaviors in male-dominated industries. This is likely due to the lack of longitudinal studies reporting pre- and post- intervention data to assess intervention effects. Help-seeking is a complex process that involves general attitudes toward seeking help, future behavioral intentions, and observable help-seeking behavior (Rickwood & Thomas, 2012). Most articles were not explicit about which part of the help-seeking process was under investigation and often referred to the outcome as “help-seeking.” It is essential that future research investigates each part of the help-seeking process and explores the strength of relationships between attitudes, intentions, and behaviors. This could help to determine where interventions have the most effect and identify more specific barriers to help-seeking within the help-seeking process.

There were limited intervention effects for help-offering intentions and behaviors. While some studies reported a significant increase in confidence around help-offering, this did not translate to any increased willingness to offer help or incidences of help-offering behavior. This may be explained by the lack of clear “help-offering” construct definitions despite its use in the wider literature (Gullestrup et al., 2023), outcome measures with poor psychometric properties, and the short-term nature of the interventions. While significant changes were observed post-intervention in the included studies, the extent to which these findings can be attributed to intervention effects is limited. Further exploratory research is needed to establish a theoretical basis and construct definition for help-offering more generally, and longitudinal studies investigating the factors influencing willingness and incidences of help-offering may benefit the field.

There were 16 BCTs identified across the interventions, the most common being “information about consequences”; “information about antecedents”; “social support”; and “credible source.” These findings are similar to previous reviews (De Korte et al., 2018; Sagar-Ouriaghli et al., 2019). Poor health literacy is associated with delayed help-seeking (Jorm et al., 2006) and is cited as a barrier to help-seeking among men (Harding & Fox, 2015). Adherence to hegemonic masculine ideology has been cited to explain delays in the help-seeking process (Cheong et al., 2020). Help-seeking can often be viewed as a sign of weakness (Lemos et al., 2017), with some men associating the tolerance of pain and illness with the preservation of masculinity (O’Brien et al., 2005). Increasing health literacy is a key factor in achieving behavior change (Mursa et al., 2022). Specifically, the use of psychoeducational intervention content incorporating the BCTs “information about consequences” and “information about antecedents” detailing the impact of mental ill-health along with risk and protective factors for mental disorders within interventions may benefit male-dominated industries where lower levels of mental health literacy have been reported (Milner et al., 2020). The BCT “credible source” leverages male role models to share personal experiences of engaging in help-seeking behavior. This has been identified as an effective way to encourage help-seeking and reduce stigma among men (Ferrari, 2016). This is often most effective when the role model is perceived to be masculine (Gough & Novikova, 2020) and interventions utilizing this BCT may reframe traditional masculine traits, such as strength and independence as positive attributes that facilitate the help-seeking process (Sagar-Ouriaghli et al., 2019).

The emphasis placed on social support across interventions aligns with existing research suggesting that signposting men to relevant services is an effective strategy for men’s health promotion (Bell et al., 2023; Robertson et al., 2018; Sagar-Ouriaghli et al., 2019). Men are reported to rely more heavily on female partners or friends for social support when experiencing mental ill-health (Oliffe et al., 2011) to maintain a masculine image in public (McKenzie et al., 2018). Interventions emphasizing the importance of social support contribute to normalizing the help-seeking process. They also foster dialogue around mental ill-health among men and challenge the perception that help-seeking compromises traditional masculine traits such as self-sufficiency (McKenzie et al., 2018). Men may prefer practical, skills-based therapies compared to “just talking” with masculine norms such a stoicism cited as a barrier to full engagement with talk therapies (Seidler et al., 2016). The incorporation of “instruction on how to perform a behavior,”“demonstration of the behavior,” and “behavioral practice and rehearsal” may provide men with action-based practical skills that they may find more acceptable.

Overall, these BCTs provide a link between research and practice when designing interventions and provide observable, replicable, and evidence-based components of an intervention that aligns with men’s health promotion research. Understanding BCTs through the lens of existing men’s health promotion strategies and masculinities theory allows for the design of tailored interventions that consider the broader context of societal expectations and gender roles. This enables a standardized approach to gender-sensitive intervention design and sheds light on components and strategies used in men’s health interventions that can be modified to align with, challenge, or reframe traditional gender norms. This approach acknowledges the impact of gender on health behaviors and provides a practical strategy for designing culturally and gender-sensitive interventions in male-dominated industries. Further research is needed to evaluate the specific role of individual BCTs in bringing about such change, and if some BCTs are more effective than others. Future research might consider greater integration of these BCTs within existing frameworks for engaging men to ensure that they are acceptable within the context of a male-dominated environment (Galdas et al., 2023; Struik et al., 2019).

There are several limitations to this review. Included papers were limited to those published in the English language, which were peer reviewed and excluded gray literature. Therefore, it is possible that papers outside these criteria may have been missed. The definition of a “male-dominated industry” in this review could be considered as too narrow in not capturing all employees in male-dominated industries. In most cases, the BCTs or “active ingredients” were not clearly articulated; therefore, BCTs may be underreported. Most studies included for review were rated as weak methodological quality, making it difficult to gauge intervention effects.

## Conclusion

Overall, there is some evidence to suggest that workplace psychosocial interventions may influence mental health literacy and help-seeking intentions. However, methodological issues and poor research design make it difficult to draw any firm conclusions from these interventions. Despite this, male-dominated workplaces provide a promising setting to go beyond gender-sensitive interventions toward more gender transformative approaches that “work with and rework” traditional masculine norms (Galdas et al., 2023). While findings are promising, the need for more robust studies is apparent. This review provides an evidence base for future intervention design through the identification of BCTs and their links to the wider men’s health literature. It is recommended that future research utilizes a standardized approach to reporting intervention content such as the TIDieR Checklist (Hoffmann et al., 2014) and utilize BCTs in conjunction with existing frameworks for men’s health interventions (Galdas et al., 2023). This provides a template to ensure all relevant information is included when describing an intervention, which may result in more rigorous identification of BCTs for future intervention design and replication.

## Supplemental Material

sj-docx-1-jmh-10.1177_15579883241236223 – Supplemental material for Workplace Interventions Targeting Mental Health Literacy, Stigma, Help-Seeking, and Help-Offering in Male-Dominated Industries: A Systematic Review Supplemental material, sj-docx-1-jmh-10.1177_15579883241236223 for Workplace Interventions Targeting Mental Health Literacy, Stigma, Help-Seeking, and Help-Offering in Male-Dominated Industries: A Systematic Review by Emilie Roche, Noel Richardson, Jack Sweeney and Shane O’Donnell in American Journal of Men’s Health

sj-docx-2-jmh-10.1177_15579883241236223 – Supplemental material for Workplace Interventions Targeting Mental Health Literacy, Stigma, Help-Seeking, and Help-Offering in Male-Dominated Industries: A Systematic Review Supplemental material, sj-docx-2-jmh-10.1177_15579883241236223 for Workplace Interventions Targeting Mental Health Literacy, Stigma, Help-Seeking, and Help-Offering in Male-Dominated Industries: A Systematic Review by Emilie Roche, Noel Richardson, Jack Sweeney and Shane O’Donnell in American Journal of Men’s Health
