# Supplementary figures and images for: Workplace Interventions Targeting Mental Health Literacy, Stigma, Help-Seeking, and Help-Offering in Male-Dominated Industries: A Systematic Review
Source: Am J Mens Health. 2024 Apr 6;18(2):15579883241236223. doi: 10.1177/15579883241236223 (PMC10998494; doi:10.1177/15579883241236223)

Supplementary File 1: OVID Medline Search String


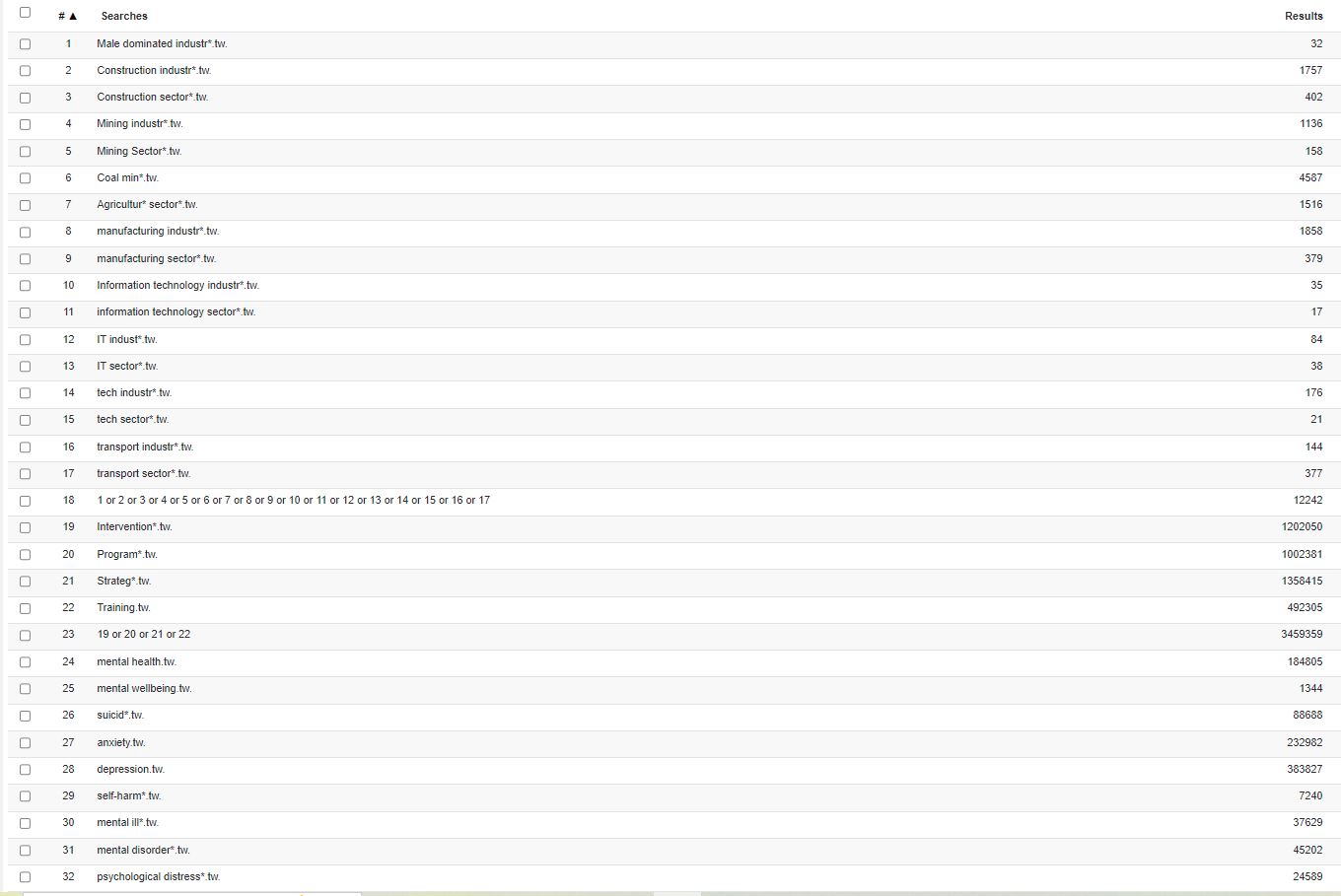

Supplement: sj-docx-1-jmh-10.1177_15579883241236223 – Supplemental material for Workplace Interventions Targeting Mental Health Literacy, Stigma, Help-Seeking, and Help-Offering in Male-Dominated Industries: A Systematic Review [file sj-docx-1-jmh-10.1177_15579883241236223.docx]
