# Supplementary material for: Workplace Interventions Targeting Mental Health Literacy, Stigma, Help-Seeking, and Help-Offering in Male-Dominated Industries: A Systematic Review
Source: Am J Mens Health. 2024 Apr 6;18(2):15579883241236223. doi: 10.1177/15579883241236223 (PMC10998494; doi:10.1177/15579883241236223)
Supplement: sj-docx-2-jmh-10.1177_15579883241236223 – Supplemental material for Workplace Interventions Targeting Mental Health Literacy, Stigma, Help-Seeking, and Help-Offering in Male-Dominated Industries: A Systematic Review [file sj-docx-2-jmh-10.1177_15579883241236223.docx]

Table 2 Characteristics of included studies.

| **Author** | **Country** | ***n***  **(% male)** | **Sector** | **Mean age in years**  **(SD)** | **Design** | **Time** | **Outcome Measure(s)** | **Intervention Description** | **Results** |
| --- | --- | --- | --- | --- | --- | --- | --- | --- | --- |
| **Gast et al., 2022** | Germany | n=70 (94.3%)  Control: n=40 | Industrial | 48.07  (8.65) | RCT | t1=baseline; t2=3months | MAKS; SoDi; PSS; ERI; IS & PHQ-4 | One day (7.5hrs) manager training to reduce stigma of mental health and improve stress-related variables of managers. | - Significant improvement in MAKS vs control at follow-up (p <0.040) - Significant difference in IS at baseline vs control (p<0.011) - No change in ERI, PHQ, SoDi or PSS |
| **Gullestrup et al., 2011** | Australia | n=7,666  ( - ) | Construction | - | Non-equivalent controlled pre/post design | t1=baseline  t2=post intervention | Ad-hoc suicide prevention awareness measure (5 pre-GAT items; t1; 5 different post-GAT items; t2);  ad-hoc SafeTalk questions (4items; t2 only) | Multilevel, peer-based suicide prevention programme. Evaluation of one-hour general awareness training (GAT); 1 day SafeTalk “Connector” component; & crisis telephone line. | - **GAT**: Significant difference in four post-GAT items vs control (p<0.001 – items relating to suicide warning signs; suicide is everyone’s business; mental health is a workplace safety issue; & need for industry to act on suicide. No significant difference on item relating to ‘people who suicide don’t want to die’. - **SafeTalk**: Strong endorsement on value of training; high degree of preparedness to talk to someone about suicide & diffusion intention. - **Crisis Line**: 1521 hrs of call |
| **King et al., 2018** | Australia | n=20,125  (92.1%) | Construction | - | One group pre/post design | t1=baseline  t2=post intervention | Ad-hoc suicide prevention awareness measure (four items); ad-hoc questions on help-seeking/ offering experiences and propensity (t2 only) | Multilevel, peer-based suicide prevention programme. Evaluation of one-hour general awareness training. | - Significant improvement in 3 suicide awareness items (suicide warning signs; mental health is a workplace safety issue; & need for industry to act on suicide, all p<0.001). Managers & professionals showed greatest change. No significant improvement on item “talking about suicide can cause suicide”. - High help-seeking (mean 3.74/5) and help-offering (4.60/5) propensity. |
| **King et al., 2023** | Australia | 1084  (86.7%)  Control face to face only:  N= 575 | Construction | - | RCT | T1=baseline  T2=3 months | suicide prevention literacy scale modified from mates (ii) help seeking intentions modified general help seeking questionnaire | Multilevel, peer-based suicide prevention programme. Evaluation of one-hour general awareness training in addition to a smart phone app | - No significant changes in suicide prevention literacy between groups - Some evidence to suggest app was more effective than face to face training in increasing participants’ intention to seek help from some sources. - No significant difference in intention to seek help from other sources. |
| **Milner et al., 2018** | Australia | n=478  (100%)  Control: n=231 | Construction | - | RCT | t1=baseline  t2=6 weeks | SSDS | Six-week online brief contact intervention where one message with hyperlinks to information on stigma and mental health literacy was sent via text message per week. | - No significant improvement in SDSS from vs control |
| **Milner et al., 2019** | Australia | n=442 (100%)  Control: n=302 | Construction | - | RCT | t1=baseline  t2=6 weeks | SBQ-R | Six week brief contact intervention where one message with hyperlinks to information on stigma and mental health literacy was sent via text message per week. | - No significant improvement in SDSS from vs control |
| **Ross et al., 2020a** | Australia | n=4,887  (73.2%) | Energy | 42.7  (11.1) | One group pre/post design | t1=baseline  t2=post intervention | GHSQ; ad-hoc emotional wellbeing question; ad-hoc suicide literacy measure (seven items relating to suicide prevention related knowledge & attitudes to help-seeking and offering) | Multilevel, peer-based suicide prevention programme. Evaluation of one-hour general awareness training. | - Significant improvement in all suicide literacy items (talking about suicide can cause suicide; warning signs; suicide is a problem in the industry; willingness to seek help; knowing where to go if in distress; noticing a colleague in distress; willingness to offer help -all p<0.001). - Significant improvement in all GHSQ items except GP (p<0.001). - Significant improvement in emotional wellbeing (p<0.001). |
| **Ross et al., 2020b** | Australia | n=2,977  (92.7%) | Construction | - | Time series | t1=baseline  t2=post intervention  T3= 3-6month follow-up | GHSQ; ad-hoc emotional wellbeing question; ad-hoc suicide awareness measure (six items relating to suicide prevention related knowledge & attitudes to help-seeking and offering) | Multilevel, peer-based suicide prevention programme. Evaluation of one-hour general awareness training (GAT); and shortened 15 minute Mates awareness training (MAT) | - Significant change in five suicide awareness items from T1 to T2 (talking about suicide can prevent suicide; noticing a colleague in distress; knowing how to connect a colleague to support; perceived support of worksite; willingness to seek help - all p<0.001). No significant change to item willingness to offer help. - Significant improvement for all GHSQ items (p<0.001) except GP from t1 to t2. Not maintained at t3. - Significant increase in emotional wellbeing from t1 to t2 (p<0.001) not maintained at t3. - No differences between GAT and MAT on any measure. |
| **Sage et al., 2016** | UK | n=50  (78%) | Transport | - | One group pre/post design | T1=baseline  T2=post-intervention | PCL-C; GHQ-12; ad-hoc questions on cohesion, leadership, mental health stigma & mental health literacy | 2-day trauma-risk management programme to improve mental health & attitudes towards mental health. | - No significant difference on PCL-C; GHQ-12 or total score for ad-hoc measures on leadership or stigma compared to baseline. Significant change in total score on ad-hoc measures for cohesion (p<0.05) and mental health literacy (p<0.001). |
| **Sayers et al., 2019** | Australia | n=1,651 (83.6%) | Mining | - | Repeated cross-sectional survey | t1=baseline; t2=6months;  t3=18months; | GHSQ; 3 items from PSS | Multilevel, peer-based suicide prevention programme. Evaluation of combined impact of one-hour general awareness training; 1-day SafeTalk component & 2-day ASIST training | - Significant change in two items relating to attitudes towards mental health across all time points (not treated differently by friends and by colleague due to mental illness (p<0.01). No change to item “not being treated poorly in workplace due to mental illness”. - Significant improvement in some GHSQ items (supervisor, colleague & EAP p<0.01; friend p<0.02; psychologist p<0.05; family member p<0.05). No significant improvement for GP, social worker, counsellor, mental health nurse or MATES connector. - Females more likely to seek help. |
| **Schwarz et al. 2019** | Germany | n=41  (85.4%) | Industrial | 47.8  (8.40) | Time series | t1=baseline; t2=3months  t3=12months | MAKS; SoDi; ERI; IS; PHQ-D; SF-12; ad hoc stigma questions | One day (7.5hrs) manager training to reduce stigma of mental health and improve stress-related variables of managers. | - Significant improvement in MAKS from t1 vs t3 (p <0.010) - Significant improvement in perceived mental health status (p=0.043) & perceived social support in workplace from T1 vs T3 (p<0.041) - No change in ERI, PHQ-D, SoDi; SF-12 or other ad-hoc stigma questions |
| **Tynan et al., 2018** | Australia | n=1,277 | Mining | - | Controlled Clinical Trial | t1=baseline  t2=post intervention | 1 item from PSS; ad-hoc questions assessing GAT & SafeTalk training; ad-hoc questions assessing manager training; frequency of use of mental health services in past 12months; | Multilevel, peer-based suicide prevention programme. Evaluation of one-hour general awareness training; 1 day Safe Talk Training; & 2hr manager training. | - **GAT & SafeTalk**: Significant improvement in all ad-hoc items within intervention group (confidence to identify a colleague in distress; willingness to talk to a colleague about mental health; confidence to identify supports; commitment of workplace to mental health; effectiveness of workplace in addressing mental health problems - all p<0.001). Did not compare to control. - **Manager Training**: significant improvement on all ad-hoc items within intervention group (p<0.001 for items confidence to identify a colleague in distress; willingness to talk to a colleague about mental health, confidence to identify supports, confidence to have effect conversations about performance issues relating to mental health; & p<0.005 for item describing the mental health continuum). Did not compare to control. - No significant difference between groups on help-seeking behaviors and PSS. |

Note: MAK=Mental Health Knowledge Schedule; SoDi=Social Distance Scale; PSS=Perceived Stress Scale; ERI= Effort Reward Imbalance; IS=Irritation Scale; PHQ4=Patient Health Questionnaire for Anxiety & Depression; PHQ-D= Patient Health Questionnaire for Depression; SF-12 = 12-Item Short Form Survey; SSDS=Self-Stigma of Depression Scale; SBQ-R= Suicidal Behavior Questionnaire Revised; GHSQ= General Help-Seeking Questionnaire; K10+ = Kessler 10 Psychological Distress Scale: PSS = Perceived Stigma Scale; JCQ= Job Content Questionnaire; GHQ-28 = General Health Questionnaire 28 item; PCL-C= PTSD Checklist Civilian; GHQ-12 = General Health Questionnaire 12-item

Table 3 Table summarizing identified intervention functions, BCTs and examples.

| **Studies** | **Intervention** | **Intervention Function** | **BCTs** | **Example(s)** |
| --- | --- | --- | --- | --- |
| Gullestrup et al., 2011; King et al., 2018; Ross et al., 2020a; Ross et al., 2020b; Sayers et al., 2019; Tynan et al., 2018; king et al., 2023 | Multilevel, Peer-Based Suicide Prevention Programme    *(i) GAT training*  *(ii) connector training (iii) manager training (iv) telephone support & counselling services* | - Education - Training - Persuasion - Modelling | 3.1. Social support [unspecified]  3.2. Social support [practical]  3.3. Social support [emotional]  4.1. Instruction on how to perform behaviour  4.2. Information about antecedents  5.1. Information about health consequences  5.3.Information about social and environmental consequences  5.6. Information on emotional consequences  6.1. Demonstration of behaviour  9.1. Credible source  11.2 Reduce negative emotions  6.3 Information about others’ approval  15.1 Verbal persuasion about capability | (i) *GAT*: increase awareness of mental health and suicide in industry; reduce stigma; encourage help-seeking and help-offering; facilitator with lived experience.  (ii) *Connector*: role of a helper; how to be alert to signs of suicide; how to ask about suicide; & how refer to help; how to look after self.  (iii) *Manager:* build confidence and skills to respond to staff experiencing mental ill-health and/or suicidal behaviour; vignette case studies to promote discussion  (iv) Telephone support and counselling services available |
| Milner et al., 2018; Milner et al., 2019 | Informational and educational brief contact intervention | - Education - Persuasion | 3.1. Social support [unspecified]  4.2. Information about antecedents  5.1. Information about health consequences  5.3.Information about social and environmental consequences  5.6. Information on emotional consequences  7.1. Prompts/cues | Text message with hyperlinks to Information on: risk/protective factors for depression and suicide; importance of social support, communication and help-seeking; and dispelling myths about mental health problems. |
| Gast et al., 2022; Schwarz et al., 2019 | Stress-management and stigma reduction manager training | - Education - Training - Modelling | 3.2. Social support [practical]  4.1. Instruction on how to perform behaviour  4.2. Information about antecedents  6.1. Demonstration of behaviour  8.1. Behavioural practice/rehearsal  2.3 Self-monitoring of behaviour  15.1 Verbal persuasion about capability. | Self-awareness exercise about early stress symptoms; psychoeducation on how to better identify thoughts, feelings and bodily reactions; discussion group on how to build resilience; discussion group on of managing employees with regard mental health and information about how to deal with such scenarios; information on improving communication skills through active listening; how to structure a mental health conversation; and role play. |
| Sage et al., 2016 | trauma-risk management intervention | - Education - Training - Modelling | 4.1. Instruction on how to perform behaviour  4.2. Information about antecedents  5.1. Information about health consequences  5.3. Information about social and environmental consequences  5.6. Information about emotional consequences | Education and role play to understand PTSD and stress reactions, how to plan peer-group led psychological risk assessment; how to interpret risk; and how to refer to appropriate supports. |
